# A review of North American Recent *Radiolucina* (Bivalvia, Lucinidae) with the description of a new species

**DOI:** 10.3897/zookeys.205.3120

**Published:** 2012-07-04

**Authors:** Elizabeth A. R. Garfinkle

**Affiliations:** 1 Santa Barbara Museum of Natural History, 2559 Puesta del Sol, Santa Barbara, California 93105, USA

**Keywords:** Lucinidae, Panamic Province, neotype, lectotype, new species

## Abstract

North American members in the genus *Radiolucina* are reviewed. A lectotype for the type species, *Radiolucina amianta*, is designated and descriptions and illustrations are provided. A description of a new species, *Radiolucina jessicae*, from the west coast of Mexico is presented. Key diagnostic species characteristics are outlined and compared among members of the genus.

## Introduction

Members of Lucinidae have been grouped and identified incorrectly in the past because of variable shell and anatomical characteristics. Since the discovery of chemosymbiosis with sulphide-oxiding bacteria in the early 1980’s, the systematics of Lucinidae has attracted more attention and many new genera and species have been described (Bouchet and Cosel 2004; Glover and Taylor 2007; Hickman 1994; Taylor and Glover 2006). Small species (less than 10 mm) have received less attention than their larger counterparts. There are currently over 400 Lucinidae species living in a variety of different habitats (Barnes and Hickman 1999; Roeselers and Newton 2012).

Dall (1901) placed *Phacoides amiantus* Dall, 1901 and *Lucina cancellaris* Philippi,1846 with the Indo Pacific subgenus *Bellucina* (now known as *Cardiolucina* Sacco, 1901). Later, Britton (1972) described *Radiolucina* as a new subgenus of *Parvilucina*, and included *Phacoides amiantus* Dall, 1901, *Lucina cancellaris* (Philippi 1846), and the fossil species *Phacoides waccamawensis* (Dall, 1903). Recent DNA results (Taylor et al. 2011) show that *Radiolucina amianta* and *Radiolucina cancellaris* are related to *Lucinisica*, not *Parvilucina* or *Cardiolucina* as was thought in the past.

While reviewing the Lucinidaeof the Panamic Province, morphological differences among specimens identified as *Radiolucina cancellaris* were discovered. After further research on the genus and examination of pertinent type specimens, a new species of *Radiolucina* was recognized herein named *Radiolucina jessicae*. The three Recent members of North American *Radiolucina* are described and illustrated.

## Materials, methods, and abbreviations

One hundred *Radiolucina* specimens from Mexico and Florida were examined. Of the 100, 20 were determined to be *Radiolucina jessicae*, two were *Radiolucina amianta*, and 78 were *Radiolucina cancellaris*. Six *Radiolucina jessicae* specimens were rehydrated in water and dish soap, and reconstituted anatomy was examined.

LACM- Natural History Museum of Los Angeles, Los Angeles, USA; NHMUK-The Natural History Museum, London, UK; SBMNH-Santa Barbara Museum of Natural History, Santa Barbara, USA; USNM-Smithsonian National Museum of Natural History, Smithsonian Institution, Washington DC, USA.

In the descriptions below, morphological characteristics outlined in Britton (1972), Taylor and Glover (2000), Cosel (2006), Glover and Taylor (2007), Taylor and Glover (2009), and Coan and Valentich-Scott (2012) have been used.

## Data resources

The data underpinning the analyses reported in this paper are deposited at GBIF, the Global Biodiversity Information Facility, http://ipt.pensoft.net/ipt/resource.do?r=radiolucina

## Taxonomy

### Genus Radiolucina

Britton, 1972

http://species-id.net/wiki/Radiolucina

Radiolucina Britton, 1972. Type species (original designation): *Phacoides (Bellucina) amiantus* Dall, 1901.

#### Description.

Shell shape subovate; maximum length: 9.0 mm, maximum height: 8.0 mm; with an average of 13 heavy radial ribs, overlain by thin commarginal lamellae that continue through interspaces, producing a reticulate pattern; posterior end thickened, posterior dorsal area often with low spines; pallial line often discontinuous broken into large and small segments; right valve hinge with two cardinal teeth, left valve hinge with one wide cardinal tooth, one anterior lateral tooth, one posterior lateral tooth.

#### Comparisons.

*Parvilucina* Dall, 1901 (type species: *Lucina tenuisculpta* P.P. Carpenter, 1864) attains a larger size and has fine radial ribs, and a short, broad anterior adductor muscle scars compared to *Radiolucina*, which has strong radial ribs and a long, narrow anterior adductor muscle scar.

*Pleurolucina* Dall, 1901 (type species: *Lucina leucocyma* Dall, 1886) has heavy commarginal lamellae with few broad, weak radial ribs compared to *Radiolucina*. It is similar to *Radiolucina* in that they both have a long, narrow anterior adductor muscle scar.

*Liralucina* Glover & Taylor, 2007 (type species: *Phacoides sperabilis* Hedley, 1909) has an average of 35 flat, radial ribs compared to *Radiolucina*,which has average 13 strong, radial ribs.

There is evidence (Coan and Valentich-Scott 2012) that *Radiolucina* dates back to the Miocene.

#### Literature.

Britton (1972), Hickman (1994), Taylor and Glover (2000), Glover and Taylor (2007), Taylor and Glover (2009), Coan and Valentich-Scott (2012).

### Radiolucina amianta

(Dall, 1901)

http://species-id.net/wiki/Radiolucina_amianta

Figures 1 , 4 , 5a

Phacoides (Bellucina) amiantus Dall, 1901: 826-827.
Parvilucina (Radiolucina) amianta . — Britton, 1972: 9-10
Lucina (Bellucina) amiantus Bretsky. — 1976: 273

#### Shell shape.

Subovate, extended anteriorly and posteriorly, length longer than height, slightly inflated; maximum length: 6.0 mm, maximum height: 6.0 mm.

#### Sculpture and color.

About 11 (n=2) non-bifurcating radial ribs, overlain by thin commarginal lamellae that continue through interspaces, producing a reticulate pattern; occasional intercalary ribs present; interspaces shallow, thin towards beak and progressively widening ventrally; anterior and posterior ends smooth with fine commarginal striae, posterior sometimes with spines of varying heights protruding from shell; inner shell margin finely crenulate; interior color tan, shiny.

#### Hinge.

Hinge plate thick, curved on either side of cardinal teeth; beaks prosogyrate; cardinal teeth small, right valve posterior tooth thin, anterior tooth thick, left valve middle tooth wide; lateral teeth large, posterior tooth vertical, anterior tooth horizontal; ligament sunken above cardinal teeth.

#### Adductor scars and pallial line.

Pallial line continuous; anterior adductor scar long, narrow, diverging from pallial line for about a quarter of its length; posterior adductor scar small, wide, pallial line joins at most ventral point.

#### Type specimens and type locality.

Dall did not designate a single specimen as the holotype. To stabilize nomenclature, I herein designate the lectotype to be the right valve (USNM 64276), which is the same specimen as figured by Dall 1901, plate XXXIX fig. 10, with the type locality of Yucatan Strait, North Atlantic Ocean (approximately 21.3°N, 86.2°W), 1170 m (Fig. 4). An additional right valve (USNM 1183662) in the original lot is a paralectotype.

**Figure 1.**
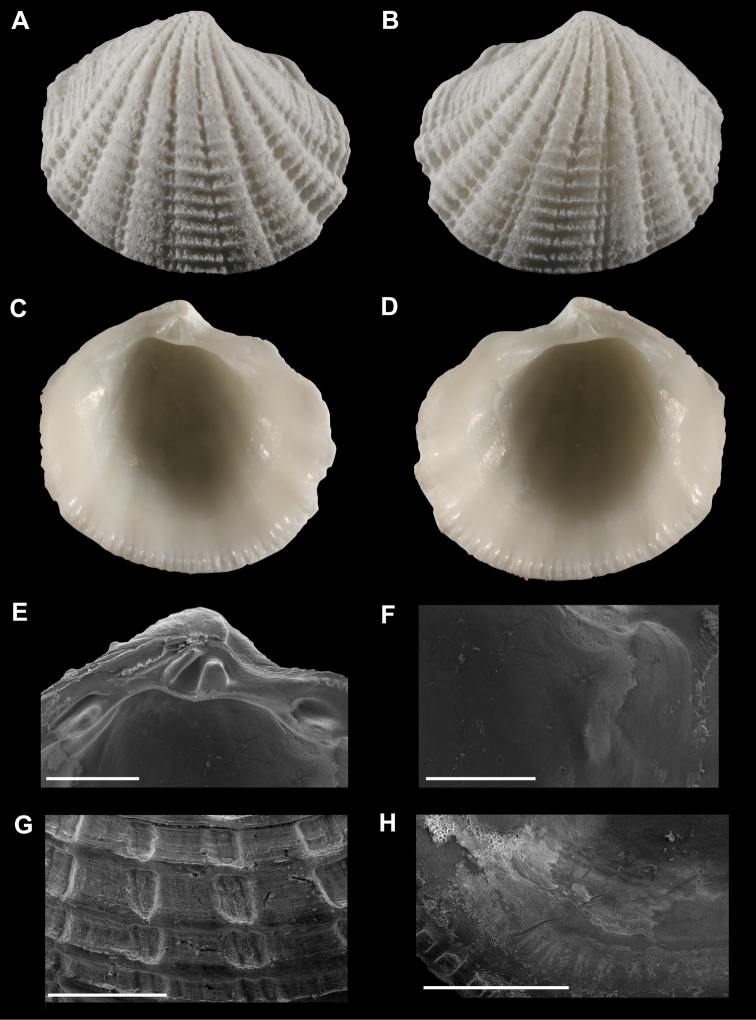
**A–D** *Radiolucina amianta* (SBMNH 357639, USA, Florida, Santa Petersburg, Tampa Bay) length = 5.4 mm **A** Exterior of right valve **B** Exterior of left valve **C** Interior of left valve **D** Interior of right valve **E** Close up of hinge of left valve **F** Close up of anterior adductor muscle scar of left valve **G** Close up of ribs of right valve **H** Close up of pallial line of left valve. **E–H** scale bar = 1 mm.

#### Distribution.

Western Atlantic from North Carolina to Florida, West Indies, Gulf of Mexico, Caribbean Central America, South America south to Uruguay (Mikkelsen and Bieler 2007).

#### Remarks.

In describing *Phacoides (Bellucina) amiantus*, Dall noted that it seemed to be the same species that Tuomey and Holmes (1857) had described as *Lucina costata* from the Pleistocene of South Carolina (non *Lucina costata* d’Orbigny, 1846). Boss et al. (1968: 25) misinterpreted Dall’s proposal as a new name, but it is expressly a new species. Moreover, it is not at all clear that these represent the same species.

#### Literature.

Dall (1901), Bretsky (1976), Mikkelsen and Bieler (2007), Tunnell et al. (2010).

### Radiolucina cancellaris

(Philippi, 1846)

http://species-id.net/wiki/Radiolucina_cancellaris

Figure 2 , 5b

Lucina cancellaris Philippi, 1846: 21.
Radiolucina cancellaris —Olsson 1961: 547.
Radiolucina cancellaris — Keen 1971: 1064.
Radiolucina cancellaris neotype — Coan and Valentich-Scott 2012: 359.

#### Shell shape.

Subovate, inflated; maximum length: 7.2 mm, maximum height: 8.0 mm; beaks prosogyrate.

#### Sculpture and color.

Average 12 (10–15 n=76) non-bifurcating radial ribs, overlain by thin commarginal lamellae that continue through interspaces, producing a reticulate pattern; interspaces sunken, thin towards beak, progressively widening ventrally; anterior end smooth, with fine commarginal striae; posterior end with a series of average 13 (12–15 n=20) thick spines of varying heights protruding from shell; inner ventral margin crenulations thin, closely spaced; interior color white to cream, shiny.

#### Hinge.

Hinge plate thick, straight with slight curve; cardinal teeth small, right valve posterior thin, anterior thick, left valve middle tooth wide; lateral teeth large, posterior vertical, anterior horizontal;ligament sunken above the cardinal teeth.

#### Adductor scars and pallial line.

Pallial line discontinuous, broken into small and large segments, with one small circular indentation directed ventrally; anterior adductor scar large, narrow, diverging from pallial line for about a quarter of its length; posterior adductor scar small, wide, pallial line joins at most ventral point.

#### Type specimens and type locality.

Neotype, SBMNH 149738 (Coan and Valentich-Scott 2012), length 6 mm, height 6 mm. Cabo Haro, Sonora, Mexico, 37–73 m.

**Figure 2.**
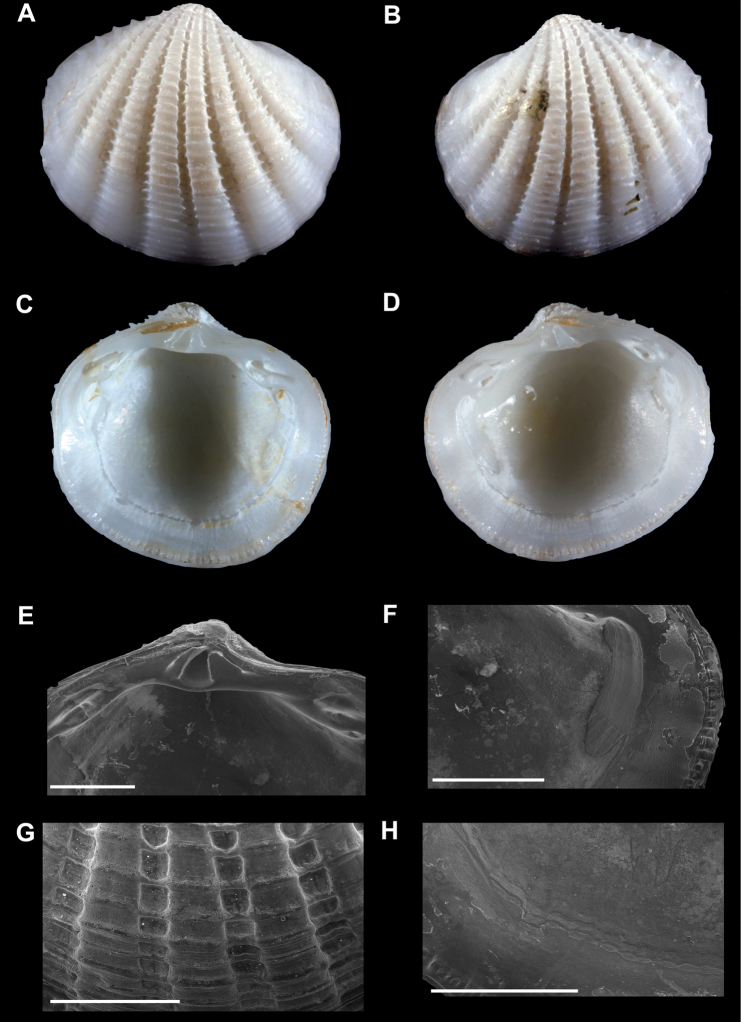
**A–D**. *Radiolucina cancellaris* neotype (SBMNH 149738, Mexico, Sonora, Cabo Haro) length = 5.5 mm **A** Exterior of right valve **B** Exterior of left valve **C** Interior of left valve **D** Interior of right valve **E–F, H** *Radiolucina cancellaris* (SBMNH 20044, Mexico, Sonora, Cabo Haro) **E** Close up of hinge of left valve **F** Close up of anterior adductor muscle scar of left valve **G** *Radiolucina cancellaris* (SBMNH 129044, Mexico, Sinaloa, Teacapan) Close up of ribs of right valve **H** Close up of pallial line of left valve. **E–H** scale bar = 1 mm.

#### Distribution.

Known from Isla Cedros, Paciﬁc coast of Baja California (28.2°N) [Keen, 1971], into the Golfo de California as far north as near its head at Puerto Peñasco, Sonora (31.3°N) [LACM], México, to Isla San Lorenzo, Lima, Perú (12.1°S) [LACM]; intertidal zone to 212 m [LACM]. Also in the Pliocene of Ecuador and the Pleistocene of Baja California.

#### Literature.

Britton (1972), Coan and Valentich-Scott (2012), Dall (1901), Keen (1971), Olsson (1961).

#### Remarks.

Shell shape and ribs vary at different stages of growth. Due to this, it can be difficult to distinguish variants of *Radiolucina cancellaris*. The number of ribs is consistent during growth (average 12); however sometimes they were thinner or thicker, and inconsistently extended to the ventral margin. Bifurcation and/or intercalary ribs are sometimes present depending on the stage of growth.

### Radiolucina jessicae

Garfinkle sp. n.

urn:lsid:zoobank.org:act:6EA53845-C2D9-4376-B460-7729CDDD3D60

http://species-id.net/wiki/Radiolucina_jessicae

Figure 3 , 5c

Radiolucina cf. *cancellaris* Coan and Valentich-Scott, 2012: 360

#### Diagnosis.

Subovate, extended anteriorly and posteriorly, slightly inflated; with about 11 primary radial ribs, excluding intercalary ribs; commarginal ribs continuing through interspaces creating a rectangular pattern; posterior end with fine commarginal striae and spines of varying heights protruding from shell; pallial line discontinuous, broken into a series of short and long sections with one large segment directed ventrally.

#### Shell shape.

Subovate, long, extended anteriorly and posteriorly, length longer than height; slightly inflated; maximum length: 6.0 mm, maximum height: 5.3 mm; beaks pointed, prosogyrate.

#### Sculpture and color.

Average 13 (9–16 n=20) radial ribs, occasional bifurcate usually on larger specimens; commarginal ribs continuing through interspaces, with 6-10 thick intercalary ribs extending to ventral edge of valve, present in most specimens, more pronounced in larger specimens; interspaces shallow and thin towards beak, progressively widening ventrally; anterior side smooth with fine commarginal striae; posterior side also smooth with fine commarginal striae, with a series of average nine (4–15 n=20) thick spines of varying heights protruding from shell; exterior color tan to white, also with brown along ribs; interior color tan, white to cream, shiny; inner shell margin crenulations thin, closely spaced.

#### Hinge.

Hinge plate thin, slightly curved on either side of cardinal teeth; right valve posterior and anterior cardinal teeth about equal in size, left valve middle tooth wide; lateral teeth large, posterior vertical and anterior horizontal; ligament long, sunken above cardinal teeth.

#### Adductor muscle and pallial scars.

Pallial line discontinuous, broken into series of large, small segments, with one large segment directed ventrally; anterior adductor scar is large, narrow, diverging from pallial line for about half its length; posterior adductor scar small, wide, pallial line joins anteriorly to most ventral point.

#### Anatomy from rehydrated dried specimens.

Inhalant aperture usually smaller than exhalant, elongate, often narrow; tissue bridge between apertures usually narrow; ventral mantle fusion thin, narrow; mantle fusion variable, usually not fused below anterior adductor muscle; rectum curves dorsally around posterior adductor muscle and ends at exhalant aperture.

#### Type locality and type specimens.

North America, Mexico, Baja California Sur, Bahía Concepción, Bahía Coyote; 26°43'50"N, 111°53'30"W; 12 m.

**Figure 3.**
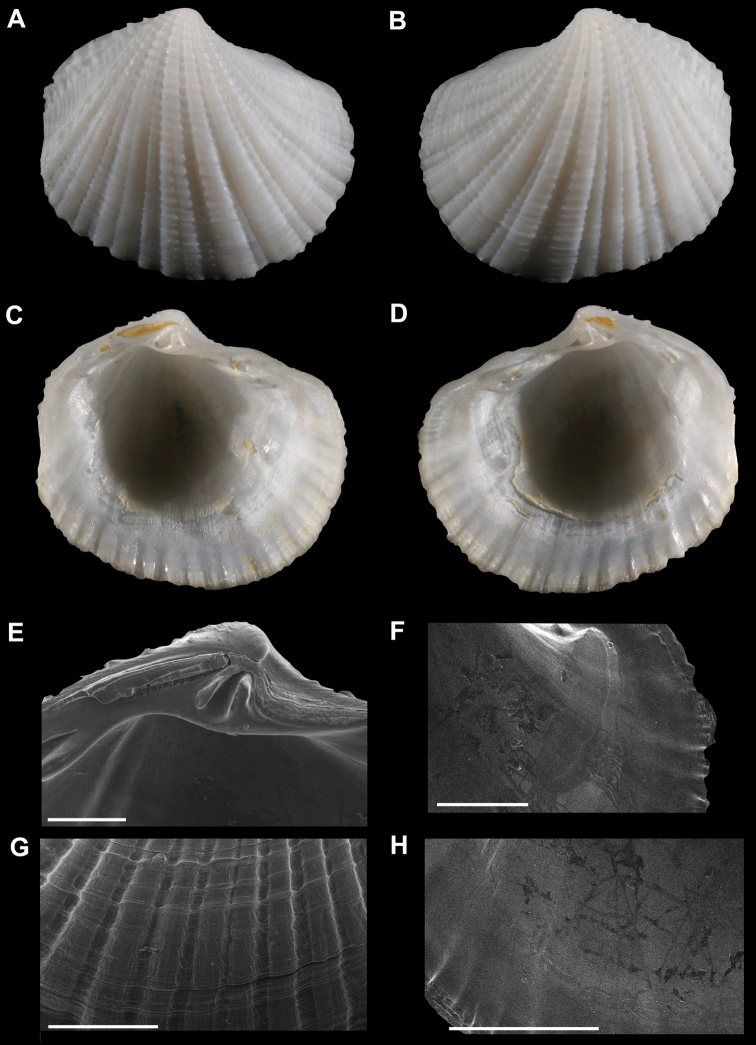
**A–D** *Radiolucina jessicae* sp. n., holotype (SBMNH 353469, Mexico, Baja California Sur, Bahia Concepcion) length = 4.5 m **A** Exterior of right valve **B** Exterior of left valve **C** Interior of left valve **D** Interior of right valve **E–H** *Radiolucina jessicae* **new species**, paratype (SBMNH 149936) **E** Close up of hinge of left valve **F** Close up of anterior adductor muscle scar of left valv **G** Close up of ribs of right valve **H** Close up of pallial line of left valve. **E–H** scale bar = 1 mm.

**Figure 4.**
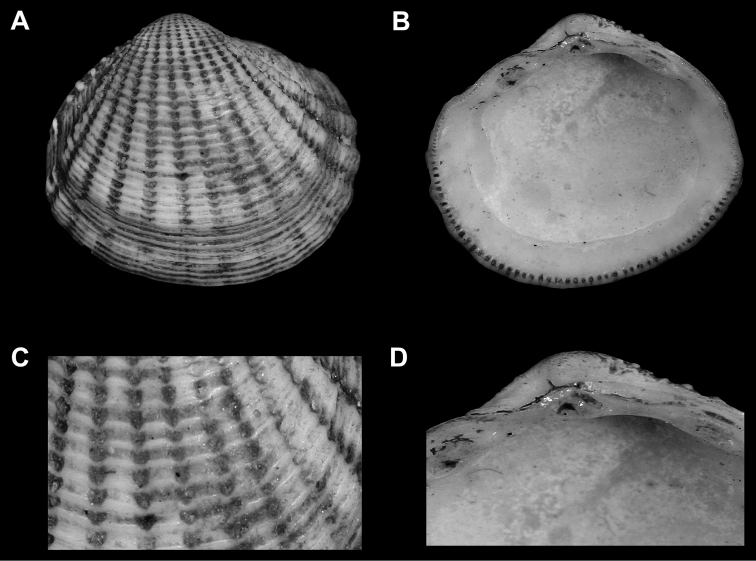
**A–D** *Phacoides (Bellucina) amiantus* Dall, 1901lectotype herein(USNM 64276, Mexico, Yucatan Strait) length = 6 mm **A** Exterior of right valve **B** Interior of right valve **C** Close up of ribs of right valve **D** Close up of hinge of right valve.

**Figure 5.**
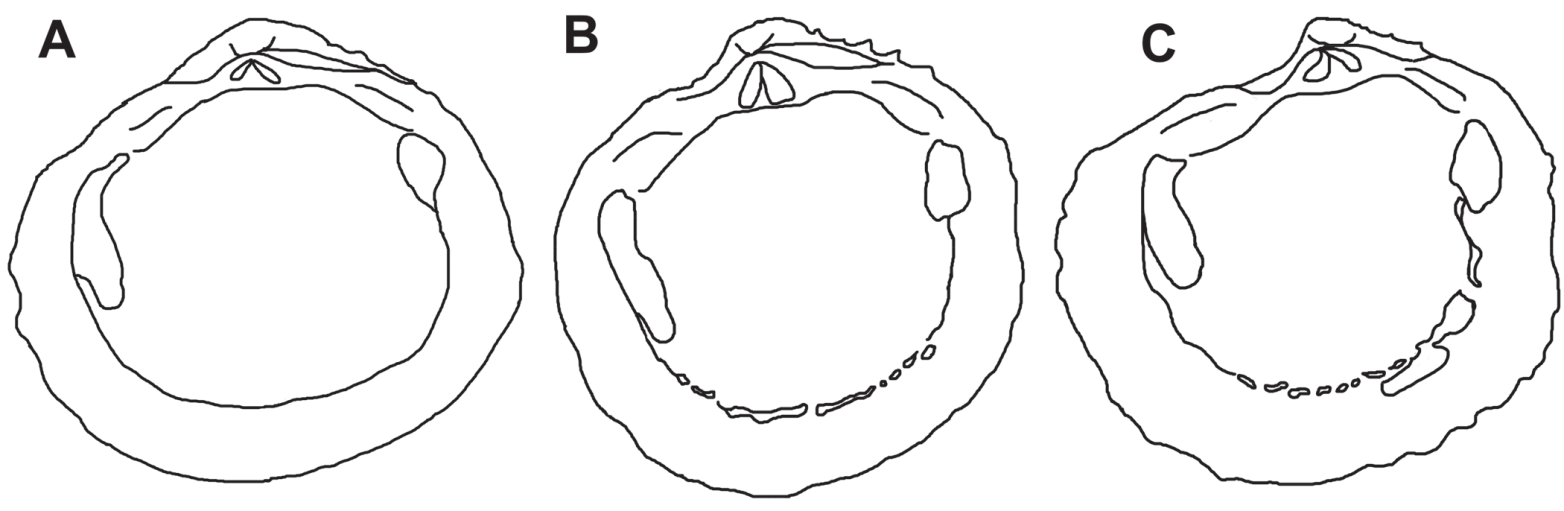
Outline drawings of interior of right valves **A** *Phacoides (Bellucina) amiantus* lectotype **B** *Radiolucina cancellaris* neotype **C** *Radiolucina jessicae* new species holotype – Not to scale.

#### Holotype.

SBMNH 353469, length: 4.5 mm. **Paratypes.** SBMNH 149936, 6 unpaired valves; LACM 3231, 4 unpaired valves; NHMUK 20120066, 2 unpaired valves; USNM 1179317, 2 unpaired valves.

#### Distribution.

East Pacific, W side of Isla El Muerto, Baja California, Mexico (30°4.00'N, 114°33.00'W) to Bahía Concepción, Baja California Sur, Mexico (26°39.00'N, 111°48.00'N). Also known from Sonora, Guaymas, Bahía San Carlos, Sonora, Mexico (27°56.1.00'N, 111°5.00'W) to San Carlos, Gulf of Panama (8°29.00'N, 79°56.00'W). Usually collected among gravel and shells; known from 13–27 m deep.

#### Etymology.

Named in honor of Jessica Sanford from Santa Barbara, California for being an inspiring scientist, meaningful mentor, and wonderful friend.

#### Comparisons.

See comparisons of *Radiolucina amianta*, *Radiolucina cancellaris*,and *Radiolucina jessicae* in Table 1.

*Radiolucina waccamawensis* (Dall 1903), from the Pliocene of the Waccamaw district, South Carolina, has about 10 strong radial ribs with deep interspaces, compared to *Radiolucina jessicae* which has about 13 radial ribs with shallow interspaces, and about eight intercalary ribs. The morphologic characters of *Radiolucina waccamawensis* are closer to *Radiolucina cancellaris*.

**Table 1.** Comparisons of key characteristics of North American *Radiolucina* species.

*Radiolucina* species	Radial ribs/interspaces	Hinge plate	Pallial line	Adductor muscle scars
*amianta*	About 11 radial, non-bifurcating with occasional small intercalary ribs;<br/> interspaces shallow	Thick, curved on either side of cardinal teeth	Continuous	Anterior long, narrow, diverging from pallial line for about a quarter of its length; posterior small, wide, pallial line joins at most ventral point
*cancellaris*	About 12 radial, non-bifurcating;<br/> interspaces sunken	Thick, straight with slight curve over entire length	Discontinuous, broken into a series of large and small segments with 1 small circular indentation directed ventrally	Anterior large, narrow, diverging from pallial line for about a quarter of its length; posterior small, wide, pallial line joins at most ventral point
*jessicae*	About 13 radial, with occasional bifurcation and thick intercalary ribs; interspaces shallow	Thin, slightly curved on either side of cardinal teeth	Discontinuous, broken into a series of large and small segments with 1 large segment directed ventrally	Anterior large, narrow, diverging from pallial line for about half its length; posterior wide, pallial line joins anterodorsally to most ventral point

#### Discussion.

Different morphologic characters among different ages of *Radiolucina cancellaris* are common, specifically ontogenetic changes in shape, rib number, and hinge teeth. Further research and use of additional characters, specifically DNA studies, (Taylor et al. 2011) could present more details and explanation for the variable characters observed, and could possibly reveal more new species within the *Radiolucina cancellaris* complex.

## Supplementary Material

XML Treatment for Radiolucina

XML Treatment for Radiolucina amianta

XML Treatment for Radiolucina cancellaris

XML Treatment for Radiolucina jessicae
